# Sick leave due to musculoskeletal disorders: A prospective cohort study

**DOI:** 10.1016/j.ijnsa.2025.100376

**Published:** 2025-07-06

**Authors:** Rigmor Harang Knutsen, Morten Birkeland Nielsen, Lars Kristian Lunde, Knut Inge Fostervold, Håkon A. Johannessen

**Affiliations:** a Research Group for Work Psychology and Physiology, National Institute for Occupational Health, 0363 Oslo, Norway; b Department of Psychology, University of Oslo, 0373 Oslo, Norway

**Keywords:** Home care services, Home health nursing, Musculoskeletal pain, Sick leave, Working Conditions

## Abstract

**Background:**

In Norway, home care workers experience particularly high levels of medically-certified sick leave. A substantial percentage of sick leave is due to musculoskeletal disorders, which may be attributed to risk factors at work. Due to limited knowledge of the impact of working conditions on sick leave in this sector, an improved understanding of occupation-specific risk factors is needed.

**Objective:**

To examine the impact of psychosocial and mechanical work factors on subsequent sick leave due to musculoskeletal disorders.

**Design:**

Prospective cohort study.

**Setting:**

A probability sample of 130 Norwegian municipalities and their respective home care services.

**Participants:**

1819 home care workers.

**Methods:**

Participants were surveyed on work environment factors and followed for 26 months between 2019 and 2021 using registry data on sick leave from the Norwegian Labour and Welfare Administration. Registry data comprised complete registrations of all medically-certified sick leave, including the relevant diagnostic codes of the International Classification of Primary Care system. Incidence risk ratios and 95 % confidence intervals were calculated using negative binomial regression with robust standard deviations. Population attributable risk and population preventable fractions were calculated to estimate the contribution of the significant work factors to sick leave in the sample.

**Results:**

The following factors were associated with excessive risk of sick leave due to musculoskeletal disorders: Lifting or supporting the patient between bed and chair or wheelchair, heavy physical exertion without aids or assistance, heavy physical exertion despite aids or assistance, walking/standing, forward bending of the upper body, squatting/kneeling, and experiencing the work as physically demanding. Control over work pacing, fair leadership, empowering leadership, and support from immediate supervisor were associated with reduced risk of sick leave.

**Conclusions:**

Preventive efforts to reduce sick leave due to musculoskeletal disorders among home care workers should adopt a multifactorial approach, including both psychosocial and mechanical work factors and the context in which they occur. There may be considerable potential for risk reduction by targeting awkward postures and heavy physical exertions, facilitating control over work pacing, and developing relationship-oriented leadership skills.

**Registration:**

Clinical Trials - NCT03855163.

**What is already known**

• The high level of sick leave among home care workers in Norway is costly, and a substantial proportion of this is due to musculoskeletal disorders.
• Both psychosocial and mechanical work environment factors are associated with increased risk of sick leave and musculoskeletal pain.
• Previous researchers have provided limited evidence of the risk factors for sick leave in the work environment of home care nurses and aides.

Alt-text: Unlabelled box

**What this paper adds**

• Awkward postures, lifting, and heavy physical exertions were mechanical factors associated with increased risk of sick leave due to musculoskeletal disorders in Norway.
• Control over work pacing and relationship-oriented leadership were psychosocial factors associated with reduced risk of sick leave due to musculoskeletal disorders.
• Based on these results, we support a multifactorial approach to prevent and reduce sick leave levels among home care workers.

Alt-text: Unlabelled box

## 1 Background

With an ageing population, there is a rising demand for eldercare workers. In addition to facing a global nurse shortage (World Health Organization, 2020), home care struggles with staff shortages, high turnover rates, and recruitment and retention of nurses (Gautun, 2021). In Scandinavian countries, the sector experiences levels of sick leave nearly two times higher than the general working population (Norwegian Labour and Welfare Administration, 2022, Solovieva et al., 2022). Sick leave is a key predictor of future disability pension and premature mortality (Gjesdal et al., 2008; Lund et al., 2008) and is associated with significant costs for the individual, employers, and society at large (Bevan, 2015). Identifying the proximal and distal causes of high sickness absence rates is, therefore, important for retaining home care employees.

Regarding proximal factors, musculoskeletal disorders are the leading cause of sick leave in Norway and the European Union (EU) (Norwegian Labour and Welfare Administration, 2022, Bevan, 2015). Musculoskeletal disorders are typically characterized by pain and limitations in functioning and have a complex and multifactorial etiology (World Health Organization, 2022). Systematic reviewers have suggested a causal relationship with age, sex, heritage, lifestyle, and work-related psychosocial and mechanical factors (Bezzina et al., 2023; Mayer et al., 2012; Taibi et al., 2021). Both psychosocial and mechanical factors are found to induce physiological stress and muscle tension, which may develop into musculoskeletal pain (Lundberg, 2002). Pain may also be due to musculoskeletal damage, for which the main cause has been identified as the influence of mechanical loads on body segments during exposure to factors, such as awkward postures and heavy lifting (Chaffin, 2009; Peters et al., 2024). This stress, acute or cumulative, leads to musculoskeletal tissue failure and subsequent tissue damage (Chaffin, 2009). Furthermore, personal characteristics (age, sex, and obesity) and psychosocial stress may also contribute to the development of musculoskeletal disorders by disrupting neuroendocrine and immune responses triggered by exposure to repeated stress (Gallagher and Barbe, 2022). This has been hypothesized to impair the healing process and reduce the fatigue resistance of musculoskeletal tissues, thus increasing the risk of injury.

Investigators conducting systematic reviews of research on musculoskeletal disorders in nurses have included studies predominantly focused on hospital or nursing home employees (Davis and Kotowski, 2015; Zare et al., 2021; Soylar and Ozer, 2018), with a paucity of studies on risk factors for musculoskeletal disorders and sick leave among home care workers. Nurses and nursing aides report musculoskeletal disorder prevalences of between 33–44 % for upper limbs (Zare et al., 2021; Davis and Kotowski, 2015) and 55 % for the low back (Davis and Kotowski, 2015). While there is a limited number of studies on home care workers specifically, pain in the neck, shoulders and lower back is highly prevalent (Andersen and Westgaard, 2014; Lundberg and Gerdle, 2017; Tjosvoll et al., 2022; Carneiro et al., 2017). However, despite home care workers’ exposure to several risk factors (Grasmo et al., 2021a) and the high levels of sick leave, there is a lack of knowledge on the impact of their unique work environment on musculoskeletal disorders and sick leave, as most previous studies have focused on pain and relied on cross-sectional designs.

Researchers investigating work factors and musculoskeletal complaints among home care workers have found associations with both psychosocial and mechanical factors. Psychosocial factors included job strain (Horneij et al., 2004), lack of influence on the planning of work (Brulin et al., 1998), emotional dissonance and being appreciated at work (Elfering et al., 2018), job satisfaction (Carneiro et al., 2017), and psychological job demands (Cheung et al., 2006). Mechanical factors have included physical demands (Andersen and Westgaard, 2014; Kim et al., 2010), awkward postures (Lohne et al., 2024; Cheung et al., 2006), patient transfers (Hsieh et al., 2022), and working with arms supported (Carneiro et al., 2017). Furthermore, Andersen and Westgaard (2014) found that mental, emotional, and physical demands were associated with perceived general tension, which, in turn, was associated with shoulder/neck and low back pain. Physical demands were also directly associated with low back pain. We were able to identify only one prospective study examining risk factors for sick leave due to musculoskeletal disorders among home care workers, performed by Horneij and colleagues (2004), who found associations with job strain and other health and leisure time factors.

Taken together, the findings recounted above emphasize the complexity of the multifactorial context in which musculoskeletal disorders can develop and, additionally, the limited knowledge of the association between work factors, pain, and sick leave within the home care sector. The present study was part of a project aspiring to gain an understanding of the role the work environment of Norwegian home care workers played in their high sick leave levels, as such insights would be valuable when developing health-promoting interventions. The aim was to identify the most prominent psychological and mechanical work factors influencing the risk of sick leave due to musculoskeletal disorders. To our knowledge, this is the first prospective study within the home care sector to measure a comprehensive set of work factors and to utilize registry data on sick leave containing medically-certified diagnostic information.

## 2 Methods

### 2.1 Design and study sample

Baseline questionnaire data on working conditions among home care workers were linked to registry data on subsequent sick leave during the 26-month follow-up period. The questionnaire data were collected as part of a project evaluating the effectiveness of the Norwegian Labour Inspection Authority’s regulatory tools for work environment and employee health in the public home care sector (Indregard et al., 2019). In Norway, municipalities are locally responsible for ensuring that all individuals—regardless of age or diagnosis—receive appropriate, high-quality health and social care services. The state, on the other hand, is tasked with establishing equitable conditions through legislation and financial frameworks, as well as overseeing supervision and regulatory control. In this study, a probability sample of 132 Norwegian municipalities and their home care services were invited to participate in March 2019. To increase statistical power for future studies on risk factors and health outcomes in this sector, eligibility criteria were expanded to include municipalities employing between 101 and 200 home care workers. Hence, an additional 48 Norwegian municipalities and their home care services were invited to participate in June 2019. This resulted in a probability sample of home care services in 180 Norwegian municipalities, of which 130 (72 %) accepted. All home care employees (7103 in total) in the participating municipalities, including home care nurses (providing professional medical care) and home care aides (assisting with personal care and housekeeping), were invited to respond to a survey questionnaire on working conditions and subjective health complaints. A total of 2591 employees participated. Of these, 1819 employees approved the linking of survey data to registry data on sick leave, yielding a final response rate of 25.6 %.

### 2.2 Measures

#### 2.2.1 Sick leave data

Norwegian employers are responsible for covering sick pay during sickness absences of 16 days or fewer, while absence of >16 days is covered by the national social insurance scheme. Sick leave data were provided by the Norwegian Labour and Welfare Administration. This constituted complete registrations on all medically-certified sick leave compensated by the social insurance system, including frequency, duration, and diagnostic codes of the International Classification of Primary Care set by the general practitioner.

We obtained sick leave data for a follow-up period of 26 months, from March 2019 through June 2021. The outcome measure constituted the count of each participant’s sick leave spells due to musculoskeletal disorders, which was defined as sick leave due to diagnoses within the musculoskeletal category of the International Classification of Primary Care system. Included in the analysis were diagnoses related to neck and shoulder complaints (codes L01, L08, L83, and L92), back complaints (codes L02, L03, L84, L85, and L86), hip and knee complaints (codes L13, L15, L78, L89, L90, and L96), and bursitis, tendinitis, synovitis, and tennis elbow (codes L87 and L93). Fractures were not included in the analysis. The sick leave data were linked to the baseline survey data through the participants’ unique 11-digit national identity number.

#### 2.2.2 Psychosocial working conditions

Psychosocial work factors were measured using validated scales from the General Nordic Questionnaire for Psychological and Social Factors at Work (Wännström et al., 2009; Dallner, 2000). The scales included in the questionnaire, followed by the Cronbach’s alpha from study results, were *quantitative demands* (α = 0.8)*, decision demands* (α = 0.72)*, learning demands* (α = 0.57)*, decision control* (α = 0.73)*, control over work pacing* (α = 0.79)*, role conflict* (α = 0.77)*, role clarity* (α = 0.79)*, support from the immediate superior* (α = 0.89)*, empowering leadership* (α = 0.88)*, fair leadership* (α = 0.78)*, predictability during the next month* (α = 0.59)*, human resource primacy* (α = 0.76)*, positive challenge at work* (α = 0.74). Each scale contains 3–5 items which measure the frequency of occurrence using the following response alternatives: 1=’very seldom or never’, 2=’seldom’, 3=’sometimes’, 4=’often’, and 5=’very often or always’. In addition, *emotional dissonance* was measured using four items from the Frankfurt Emotion Work Scales (α = 0.86) (Zapf et al., 1999). Response alternatives were 1=’seldom or never’, 2=’once per week’, 3=’once per day’, 4=’several times per day’, and 5=’several times an hour’.

#### 2.2.3 Mechanical working conditions

Mechanical work factors were measured using a total of 10 items. The first five items were developed by Statistics Norway (Statistics Norway, 2023). Time spent *squatting/kneeling, walking/standing,* and positioned with *forward bending of the upper body without support of the arms* was measured with the response alternatives 1=’never’, 2=’very small amount of the time’, 3=’a quarter of the time’, 4=’half the time’, 5=’three-quarters of the time’, and 6=’almost all the time’. Frequency of *awkward lifting* and *heavy lifting* (>10 kilos) was measured with the response alternatives 1=’never’, 2=’1–4 times per day’, 3=’5–9 times per day’, 4=’10–19 times per day’, 5=’at least 20 times per day’. To ensure that the mechanical factors were relevant for home care workers specifically, four items were adapted from Smedley et al. (1995). The items measured how often workers must 1) *manually move patients around on a bed, chair, or wheelchair,* 2) *manually lift or support patients between a bed and a chair or wheelchair,* 3) *perform heavy physical exertions without the use of aids or assistance,* and 4) *perform heavy physical exertions without aids or assistance – despite their availability*. Response alternatives 1=’never’, 2=’1–4 times per day’, 3=’5–9 times per day’, 4=’10–19 times per day’, 5=’at least 20 times per day’. Lastly, the final item included, based on Borg (1990), asked the respondents to rate perceived physical intensity of their work on a scale from 0=’not at all’ to 10=’extremely heavy’.

#### 2.2.4 Covariates

All analyses were initially adjusted for self-reported age, sex, years of education, percentage of full-time equivalent position, and time spent with patients.

### 2.3 Statistical analysis

#### 2.3.1 Negative binomial regression analyses

The relationships between psychosocial and mechanical work factors and medically-certified sick leave spells due to musculoskeletal disorders were examined using negative binomial regression analysis. Sickness absence data has frequently been analyzed using Poisson regression, but this is a form of count data and is often characterized by the variance being larger than the mean (Christensen et al., 2007). This overdispersion goes against the assumption of Poisson models that the variance is equal to the mean. Furthermore, sickness absence data tends to include more zero-values that indicate no absence, thus being zero-inflated. To account for this, we used a negative binomial model, which is a generalization of the Poisson model that is better suited to handle overdispersion. We performed negative binomial regression with robust standard errors to estimate incidence rate ratios (IRR) of the association between predictors and outcome measures. Robust standard errors are robust against heteroskedasticity (unequal distribution of error terms) and misspecification of the model. All predictors were analyzed separately with adjustment for covariates. Statistical analyses were performed using STATA 17.0 (StataCorp, 2021).

#### 2.3.2 Dominance analysis

Dominance analysis was performed to determine the relative importance of the statistically significant psychosocial and mechanical work factors to the outcome. Dominance analysis is a useful method for comparing a large number of intercorrelated predictors that collectively explain a substantial amount of variance in the outcome (Budescu, 1993). For the purpose of this analysis only, two mechanical scales were constructed to be able to compare mechanical and psychosocial factors. A scale for *general physical exertion* was generated by averaging the following items: *squatting/kneeling, walking/standing, forward bending of the upper body,* and *experiencing the work as physically demanding.* The last item was recoded from a 11-point scale to a 5-point Likert scale before inclusion. The second scale for *heavy lifting and moving of patients* was generated by averaging the following items: *lift/support patients between bed and chair/wheelchair, heavy physical exertion without aids or assistance, and heavy physical exertion despite aids or assistance.* The dominance analysis was performed using the DOMIN add-on module (StataCorp, 2021).

#### 2.3.3 Population attributable risk and population preventable fraction estimation

Another analysis was performed to indicate the contribution of the statistically-significant work factors to sick leave in the sample. The significant factors from the adjusted models in Table 2, Table 3 were dichotomized and analyzed in an additional negative binomial regression analysis. The psychosocial factors and the mechanical factor *experiencing the work as physically demanding* were dichotomized at the median level. The remaining mechanical factors were split between little/not exposed (responding 1 or 2) and exposed (responding 3 or more). We then calculated population attributable risk (PAR) estimates for risk factors (IRR > 1) and population preventable fraction (PPF) estimates for protective factors (IRR < 1). PAR is an estimate of the proportion of sick leave cases in the population that is attributable to a particular predictor. PAR and confidence intervals (CIs) were calculated as described by Natarajan et al. (2007). PAR was calculated using the formula (Pd × (IRR – 1/IRR)), where Pd is the proportion of cases exposed to the predictor (i.e., cases with scores above the median), and IRR is the adjusted IRR for sick leave due to musculoskeletal disorders. The upper and lower limits of the 95 % CI were calculated using the upper and lower limits of the 97.5 % CI for Pd and IRR, which represents the 95 % Bonferonni CI for PAR.

The proportion of cases in the population that were prevented due to the exposure to a protective factor is estimated by the PPF. As recommended by Strain et al. (2020) when potential confounders are present, the estimate was calculated using the formula (Pd(1-IRR))/(1-(1-IRR)(1-Pd)). Also as described by Strain and colleagues, the upper and lower limits of the 95 % CI were calculated by means of simulation. A normal distribution was assumed for both Pd (mean = 0.517, standard deviation [SD] = 0.041) and for the natural logarithm of the incidence rate ratio (ln[IRR]) (mean = −0.398, SD = 0.19), and 1 million values of each were simulated. We then computed 1 million values of PPF using the formula above, utilizing the simulated Pd and ln(IRR) values, which were then considered to represent the probability distribution of PPF. The 2.5th and 97.5th percentiles of this distribution represent the 95 % CIs of the PPFs.

### 2.4 Ethics approval and consent to participate

The study was conducted in line with the principles of the Declaration of Helsinki, and assessed by the Regional Committees for Medical and Health Research Ethics (REC South East), which concluded that the study was not considered medical research. The study used human participants who gave informed consent to participate in advance. Data handling and storage were approved by the Norwegian Centre for Data Research (566,128).

## 3 Results

Characteristics of the study sample are presented in Table 1. Mean age of participants was 45.5 (SD 11.86). During the 26 months long follow-up period, 773 participants (42.5 %) experienced at least one period of sick leave due to any cause. Of these, 270 of the participants (34.0 %) experienced one or more sick leave spells due to musculoskeletal disorders. Among participants experiencing musculoskeletal complaints, 38.1 % to 58.3 % reported that their pain was exacerbated by work (dependent on pain site).

**Table 1:** Characteristics of the study sample.

Variables	Total		No medically-certified sick leave		Medically-certified sick leave due to musculoskeletal disorders	
	*n*^a^	( %)	*n*	(Cases %^b^)	*n*	(Cases %^c^)
*Age*						
<30	242	13.3	139	57.4	24	9.9
30–39	342	18.8	192	56.1	47	13.7
40–49	477	26.2	270	56.6	71	14.8
50–59	520	28.6	297	57.1	89	17.1
>59	238	13.1	148	62.2	39	16.4
*Sex*						
Female	1734	95.3	987	56.9	259	14.4
Male	85	4.7	59	69.4	11	12.9
*Work status*						
Permanent employment	1716	94.9	983	57.3	254	14.8
Temporary contract	38	2.1	23	60.5	6	15.8
Substitute/extra	52	2.9	33	63.5	7	13.46
Other	3	0.2	1	33.3	1	33.3
*Supervisory position*						
Top manager	274	15.3	112	40.9	58	21.2
Middle manager	151	8.4	106	70.2	12	8.0
No management responsibility	1364	76.2	810	59.4	196	14.4
*Working with patients*						
No contact	10	0.6	8	80.0	1	10.0
Less than half the time	260	14.3	167	260	31	11.9
Half the time or more	1532	84.2	862	56.3	237	15.5

a n represents the number of observations.

b Percentage within each category with no medically-certified sick leave.

c Percentage within each category with medically-certified sick leave due to musculoskeletal disorders. Sick leave due to other diagnoses is not displayed in the table.

### 3.1 Psychosocial working conditions

Means, SDs and the prospective associations between psychosocial factors at baseline and sick leave during follow-up are presented in Table 2. In the final model, *control over work pacing, fair leadership, empowering leadership*, and *support from immediate supervisor* were associated with a decreased risk of medically-certified sick leave due to musculoskeletal disorders.

**Table 2:** Mean and standard deviations (SD) for psychosocial work factors, and incidence rate ratio (IRR) and confidence intervals (CI) for sick leave due to musculoskeletal disorders.

	Descriptive		Crude		Adjusted^a^	
	Mean	SD	IRR	95 % CI	IRR	95 % CI
Quantitative demands	3.04	0.78	0.98	(0.83–1.15)	1.15	(0.96–1.39)
Decision demands	3.63	0.66	1.07	(0.87–1.33)	1.23	(0.99–1.53)
Learning demands	2.56	0.60	0.90	(0.73–1.12)	1.00	(0.80–1.25)
Role clarity	4.30	0.66	1.14	(0.91–1.43)	0.99	(0.78–1.25)
Role conflict	2.68	0.82	1.08	(0.92–1.26)	1.14	(0.96–1.34)
Decision control	2.80	0.71	0.89	(0.73–1.08)	0.88	(0.73–1.07)
Control over work pacing	2.33	0.86	0.83	(0.70–0.99)*	0.83	(0.68–1.00)*
Positive challenge at work	4.21	0.65	1.04	(0.85–1.27)	0.99	(0.80–1.22)
Emotional dissonance	2.36	0.94	1.05	(0.91–1.21)	1.05	(0.91–1.21)
Fair leadership	4.02	0.85	0.89	(0.76–1.04)	0.85	(0.73–1.00)*
Empowering leadership	3.22	1.05	0.86	(0.76–0.98)*	0.86	(0.76–0.99)*
Support from immediate supervisor	3.80	1.02	0.90	(0.79–1.02)	0.88	(0.77–1.00)*
Human resource primacy	2.98	0.98	0.95	(0.84–1.09)	0.94	(0.83–1.08)
Predictability next month	3.47	0.94	0.92	(0.80–1.06)	0.88	(0.76–1.02)

a Adjusted for age, sex, education, percentage of full-time equivalent position, and working time spent with patients.

⁎ *p* < 0.05.

### 3.2 Mechanical working conditions

Means, SDs, and the prospective associations between mechanical factors at baseline and sick leave during follow-up are presented in Table 3. In the final model, *lifting or supporting the patient between bed and chair or wheelchair, heavy physical exertion without aids or assistance, heavy physical exertion despite aids or assistance, walking/standing, forward bending of the upper body, squatting/kneeling*, and *experiencing the work as physically demanding* were associated with an increased risk of medically-certified sick leave due to musculoskeletal disorders.

**Table 3:** Mean and standard deviations (SD) for mechanical work factors, and incidence rate ratios (IRR) and confidence intervals (CI) for sick leave due to musculoskeletal disorders.

	Descriptive		Crude		Adjusted a	
	Mean	SD	IRR	95 % CI	IRR	95 % CI
Squatting/kneeling	2.79	1.10	1.16	(1.03–1.30)*	1.16	(1.02–1.32)*
Walking/standing	4.52	1.30	1.20	(1.08–1.34)⁎⁎	1.19	(1.06–1.35)*
Forward bending of the upper body	3.06	1.27	1.20	(1.09–1.32)⁎⁎	1.17	(1.06–1.30)*
Awkward lifting	2.05	0.72	1.22	(1.03–1.45)*	1.20	(1.00–1.45)
Lifting >10 kgs	1.98	0.80	1.11	(0.95–1.31)	1.11	(0.94–1.31)
Manually move patient in bed/chair	2.02	0.73	1.17	(0.97–1.42)	1.18	(0.97–1.43)
Lift/support patients between bed and chair/wheelchair	2.09	0.75	1.31	(1.10–1.57)*	1.35	(1.13–1.62)⁎⁎
Heavy physical exertion without aids or assistance	1.77	0.67	1.28	(1.06–1.55)*	1.26	(1.03–1.53)*
Heavy physical exertion despite aids or assistance	1.53	0.64	1.26	(1.03–1.53)*	1.26	(1.03–1.54)*
Experiencing the work as physically demanding	3.77	2.12	1.15	(1.09–1.22)⁎⁎	1.15	(1.08–1.23)⁎⁎

a Adjusted for age, sex, education, percentage of full-time equivalent position, and working time spent with patients.

⁎ *p* < 0.05.

⁎⁎ *p* < 0.001.

### 3.3 Dominance analysis

Dominance statistics and rankings are displayed in Table 4. The dominance analysis showed that *general physical exertion* had the largest relative relationship with sick leave due to musculoskeletal disorders and accounted for the majority of the predicted variance in the outcome. Mechanical factors accounted for 71.6 % of the predicted variance, and the psychosocial factors in total accounted for 28.4 %.

**Table 4:** Results of dominance analysis showing rank of relationships with sick leave due to musculoskeletal disorders.

Variable	Dominance statistics	Standardized dominance statistics	Ranking
General physical exertion	0.0060	0.5420	1
Heavy lifting and moving of patients	0.0019	0.1741	2
Control over work pacing	0.0013	0.1196	3
Empowering leadership	0.0012	0.1053	4
Support from immediate supervisor	0.0004	0.0342	5
Fair leadership	0.0003	0.0249	6

### 3.4 Population attributable risk and population preventable fraction estimates

PAR estimates were calculated to quantify the proportion of sick leave spells due to musculoskeletal disorders that could be explained by the risk factors that emerged in the previous analyses. PPF estimates were calculated to quantify the protective factors’ contribution to preventing sick leave spells due to musculoskeletal disorders in this sample. Risk and protective factors were dichotomized and reanalyzed for the purpose of the PAR and PPF calculations. PAR and PPF estimates are presented in Table 5. *Experiencing the work as physically demanding, forward bending*, and *squatting/kneeling* were estimated to explain the largest respective proportions of sick leave in the sample.

**Table 5:** Population attributable risk (PAR) and population prevented fraction (PPF) of sick leave due to musculoskeletal disorders.

Variable^a^	*n*^b^	Cases %^c^	Median^d^	IRR (95 % CI)^e^	PAR (95 % CI)
Squatting/kneeling	1799	54.0	–	1.60 (1.20–2.14)*	23.9 % (7.54–38.70)*
Walking/standing	1796	91.7	–	1.88 (0.83–4.25)	44.7 % (−32.54–77.75)
Forward bending of the upper body	1791	62.3	–	1.68 (1.24–2.29)*	29.6 % (10.44–45.94)*
Lift/support patient between bed and chair/wheelchair	1807	21.8	–	1.49 (1.08–2.07)*	8.1 % (0.50–16.34)*
Heavy physical exertion without aids or assistance	1798	8.7	–	1.42 (0.90–2.25)	3.0 % (−1.14–8.30)
Heavy physical exertion despite aids or assistance	1794	5.0	–	1.58 (0.88–2.85)	1.9 % (−0.53–5.67)
Experiencing the work as physically demanding	1812	52.9	4	2.01 (1.51–2.68)⁎⁎	33.1 % (18.47–46.44)*

					PPF (95 % CI)

Control over work pacing	1795	55.3	2.3	0.76 (0.58–1.01)	13.4 % (−0.00–0.26)
Fair leadership	1806	64.0	4	0.85 (0.64–1.13)	9.6 % (−0.08–0.26)
Empowering leadership	1798	50.8	3.3	0.73 (0.56–0.96)*	14.0 % (0.02–0.26)*
Support from immediate supervisor	1783	54.4	4	0.81 (0.65–1.11)	10.5 % (−0.03–0.24)

a Factors significant in the adjusted analysis in Table 2, Table 3.

b N represents the number of observations.

c Participants with scores above the median.

d Median value for factors dichotomized at median level.

e Incidence rate ratios (IRR) and confidence intervals (CI) adjusted for age, sex, education, percentage of full-time equivalent position, and working time spent with patients.

⁎ *p* < 0.05.

⁎⁎ *p* < 0.001.

## 4 Discussion

We aimed to identify the most prominent psychosocial and mechanical work factors influencing risk of medically-certified sick leave spells due to musculoskeletal disorders among Norwegian home care workers. To our knowledge, this is the first study utilizing a prospective design and registry data with diagnostic information to investigate sick leave due to musculoskeletal disorders among home care workers. A total of 14 psychosocial factors and 10 mechanical factors were examined, of which four psychosocial and seven mechanical factors were significantly associated with risk of sick leave. The mechanical factors explained the majority of the predicted variance in the outcome (71.6 %), and the psychosocial factors accounted for the remaining 28.4 %.

The significant mechanical work factors were in line with previous studies suggesting an association between physical demands, forward bending, and transferring of patients and musculoskeletal complaints among home care workers (Andersen and Westgaard, 2014; Carneiro et al., 2015) and nurses (Soylar and Ozer, 2018). We have identified a substantial potential for reducing risk of sick leave by targeting awkward postures, such as forward bending without the support of arms (PAR = 29.6 %), squatting/kneeling (PAR = 23.9 %), and heavy physical exertions involving lifting. This may, in turn, reduce the extent to which workers *experience the work as physically demanding* (PAR = 33.1 %).

The significant psychosocial work factors are in line with previous researchers who suggested that low support at the workplace was associated with musculoskeletal disorders among nurses in general (Zare et al., 2021; Bernal et al., 2015) and another study on healthcare workers, in which researchers found that autonomy and high levels of social support were protective factors against musculoskeletal disorders (Keyaerts et al., 2022). Moreover, factors such as less social community and influence at work and more time in manual work are associated with increasing levels of sick leave due to musculoskeletal pain over time (Hallman et al., 2019). *Control over work pacing* was the highest ranked psychosocial factor in the dominance analysis and thus the most influential in reducing the risk of sick leave due to musculoskeletal disorders. Workers’ influence over the timing and duration of their breaks may contribute to reducing general tension or psychological strain (Lundberg, 2002), factors that may mediate the relationship between psychosocial factors and musculoskeletal disorders (Andersen and Westgaard, 2014; Eatough et al., 2012). This facet of job control may, therefore, be important to prevent pain development and exacerbation of existing conditions to the point where job demands exceed work capacity and sick leave is necessitated.

Fair, empowering, and supportive leadership can be described as elements of a relationship-oriented approach to leadership. This type of approach was found to lead to more positive results in a nursing context when compared with task-oriented leadership styles on a range of outcomes, including mental health, job satisfaction, work environment, and productivity (Cummings et al., 2010). Emotionally-skilled leaders were able to understand workers challenges, be supportive, and invest in their workers, which, in turn, facilitated task accomplishment and could have a direct positive impact on patient outcomes. Furthermore, Rasmussen et al. (2022) found that eldercare managers’ knowledge of preventive measures against musculoskeletal pain and injuries and communication of workers’ entitlements to assistance with pain management or accommodations were associated with future pain and sickness absence. Leadership factors are also likely to contribute to the level of control workers have over their work pacing and the suitable organization and distribution of the workload within a team or unit. Thus, investing in managers’ relational skills and knowledge of prevention of and accommodation for musculoskeletal disorders may positively affect home care workers health and well-being.

Cumulative exposure to psychosocial and mechanical stressors can lead to musculoskeletal complaints and subsequent sick leave (Chaffin, 2009; Coenen et al., 2014; Ford et al., 2014), and researchers have emphasized an additive effect of combinations of adverse psychosocial and mechanical factors on risk of sick leave (Helgesson et al., 2020; Sterud et al., 2024). Effects of exposures are, in other words, expected to develop over some time. It is, therefore, not surprising that the mechanical factors in our analysis emerged as the most dominant predictors of sick leave due to musculoskeletal disorders, but stronger effects of psychosocial factors may have been observed if the follow-up period had been longer than 26 months. However, it is uncertain what the optimal time lag was for our outcome (Ford et al., 2014). It has also been suggested that an effect of psychosocial factors cannot be identified when there is a high risk of musculoskeletal disorders due to high mechanical workload (Hauke et al., 2011).

Managing the work environment is central to the prevention of musculoskeletal disorders. The conditions in which musculoskeletal disorders arise are created by shared work organization factors, such as time pressure and colleagues’ attitudes and behaviors, which shape workers’ perception of and reaction to work events (Faucett, 2005). Home care workers have previously been found to prioritize patient care over ergonomics and, in the process, devalue their personal safety (Carneiro et al., 2015). Workers have also reported experiencing individualization of the responsibility for creating healthy working conditions (Grasmo et al., 2021a), as well as hesitation to prioritize personal health and safety, if doing so will be at the expense of their patients or coworkers. Such mechanisms may develop from work organization factors and help explain why Tjosvoll et al. (2022) found a considerable individual variation in exposure to mechanical factors among home care workers.

Furthermore, while transfer devices, particularly motorized lifting equipment, have been found to significantly reduce biomechanical stress and risk of musculoskeletal disorders among nurses (Abdul Halim et al., 2025; Abdul Halim et al., 2023), required equipment for transfers or mobility may be lacking in home care (Grasmo et al., 2021b; Pettersson et al., 2020). Workers have reported that implementation of such aids is a major challenge due to the physical environment of ordinary homes, where narrow spaces and physical barriers prevent utilization of equipment and safe work techniques (Pettersson et al., 2021) Additionally, organizational factors that are prevalent in the home care sector, such as time pressure, staff shortages, and high workload, have been recognized as significant barriers for use of patient lifts despite their presence (Khairallah et al., 2024). Finally, the physical environment also contributes substantially to care provision in awkward postures, including squatting/kneeling and forward bending (Grasmo et al., 2021a), exposures that were estimated to explain notable proportions of sick leave spells in the present study. Home care services must adopt a holistic approach to their work environment, as demonstrated by Albanesi et al. (2022), who found that multifactorial interventions have shown significant reduction in work-related musculoskeletal injuries and pain among healthcare workers. Despite these insights, interventions aiming to reduce musculoskeletal disorders mainly focus on mechanical risk factors in the workplace, thus bypassing the acknowledged potential impact of psychosocial factors (Macdonald and Oakman, 2015; Albanesi et al., 2022).

The high PAR estimates in the present study may be explained partly by a direct effect of work environment on sick leave and partly by reverse causation. A cumulative effect of risk factors can lead to reduced work ability that may be exceeded by job demands and thus necessitate sick leave. The cumulative exposure to chronic stressors can also be expected to increase the reactions to those stressors (Ford et al., 2014). Furthermore, the effect of work environment on work ability may also be substantially stronger in vulnerable sub-populations with poor health (Burr et al., 2022) and thus exacerbate pre-existing conditions and contribute to higher risk of sick leave. In the interest of retaining workers with reduced work ability, further research on work factors moderating the relationship between musculoskeletal complaints and sick leave among home care workers is warranted.

### 4.1 Strengths and limitations

The main strengths of this study are the prospective study design, probability sample, and diagnosis-specific registry data on sick leave. The measures of the predictor and outcome variables were collected from different sources, and thus there is no risk of common method bias (Podsakoff et al., 2003). There was no attrition during follow-up and little risk of reporting bias in the outcome variable due to the use of registry data. The diagnostic information included in the sick leave data reduced the risk of non-differential measurement errors. Robust standard errors were applied in the analysis to account for heteroscedasticity and multiple testing, and the estimates yielded must be considered conservative. Thus, the significant associations are unlikely to be a result of a Type 1 error. Conversely, as a result, it is possible that some true effects went undetected.

The study’s lack of data on previous musculoskeletal diagnoses limited our ability to ascertain whether the results suggest that the psychosocial and mechanical work factors can predict the onset of sickness absence or indicate an increased likelihood of recurrence of sick leave due to musculoskeletal disorders. Furthermore, the analyses were not adjusted for diagnosed or potential undiagnosed mental health comorbidities, which may impact the work ability of people with chronic musculoskeletal pain (Olaya-Contreras and Styf, 2013).

The predictor variables may be subject to reporting biases, as they were measured using a self-report questionnaire. Objective measurement of factors of a subjective nature is, however, challenging. Steps were taken to limit issues relating to self-report measures, such as common method variance, subjective interpretations, and response set tendencies (Spector, 2006), by, for example, ensuring anonymity, using varying response anchors, and separating independent and dependent variables in the survey (Podsakoff et al., 2003). The psychosocial factor scales used were from the validated General Nordic Questionnaire for Psychological and Social Factors at Work, in which the items were constructed by avoiding terms with positive or negative connotations to reduce risk of confounding by reporting behaviour and rather focus on situation prevalence (Dallner, 2000). Self-reported exposures of psychosocial factors may not accurately reflect the objective work environment, but the subjective perceptions have been argued to be more important (Niedhammer et al., 1998). When measuring mechanical exposures, however, the questionnaire approach has shown varying validity (Barrero et al., 2009) and tends to overestimate the duration of activities compared to objective instruments, such as accelerometers (Gupta et al., 2017; Koch et al., 2016). Nevertheless, objective assessment of mechanical factors was not feasible for the present sample size.

We consider the study to possess external validity due to the sampling strategy. The results are largely generalizable to the public home care services that were not included in the sample and to nurses and nurses’ aides in other parts of the health and social sector. There may, however, be only limited generalizability to national contexts outside of the Nordic countries due to their unique sick leave and disability benefit systems. The final limitation is that the baseline response rate of 26 % is rather low. While there is a risk of introducing nonresponse bias, some authors have emphasized that any association between nonresponse bias and response rate is likely to be small (Hendra and Hill, 2019; Phillips et al., 2016). Furthermore, any effects the response rate may have on intra-variable associations are likely to be negligible (Beehr et al., 2022).

The present study design did not allow for inferences regarding causal relationships between the variables or constellations of variables, and we have not investigated possible confounding, interaction or mediation effects. This would be an interesting avenue of future research, in addition to intervention approaches to preventing musculoskeletal disorders and maintaining work ability of people suffering from musculoskeletal pain.

## 5 Conclusion

From our prospective study’s findings on Norwegian home care workers, we suggest that both psychosocial and mechanical work factors were associated with the risk of medically-certified sick leave due to musculoskeletal disorders. Four psychosocial factors were found to be protective factors reducing the risk of sick leave. Furthermore, seven mechanical factors relating to awkward postures, heavy physical exertion, and patient lifting increased the risk of sick leave spells due to musculoskeletal disorders. We have identified a substantial potential for reduction of sick leave within home care by working proactively to improve the organization, distribution, and implementation of work tasks and encourage relationship-oriented leadership approaches. Assistive equipment must be provided to reduce biomechanical strain whenever possible, but innovative approaches to reducing strain are required where implementation of equipment is not feasible.

## CRediT authorship contribution statement

**Rigmor Harang Knutsen:** Writing – review & editing, Writing – original draft, Formal analysis. **Morten Birkeland Nielsen:** Writing – review & editing. **Lars Kristian Lunde:** Writing – review & editing. **Knut Inge Fostervold:** Writing – review & editing. **Håkon A. Johannessen:** Writing – review & editing, Supervision, Methodology, Investigation, Funding acquisition, Conceptualization.

## Declaration of competing interest

The authors declare that they have no known competing financial interests or personal relationships that could have appeared to influence the work reported in this paper.

## Appendix Supplementary materials

Image, application 1
