# Supplementary material for: Sick leave due to musculoskeletal disorders: A prospective cohort study
Source: Int J Nurs Stud Adv. 2025 Jul 6;9:100376. doi: 10.1016/j.ijnsa.2025.100376 (PMC12369440; doi:10.1016/j.ijnsa.2025.100376)
Supplement: Supplementary file 1 [file mmc1.doc]

**IJNS author checklist** *V2024.1*

| **IJNS AUTHOR CHECKLIST:** You will need to submit a completed version of this checklist plus the checklist from any relevant reporting guideline along with your paper. It is intended to help you to make sure your manuscript meets some basic requirements for the journal. It should be read in conjunction with the guide for authors, and is not a replacement for it. Additionally, to help ensure your manuscript is compliant, a manuscript template is available (linked from the guide for authors). | | Insert a tick, page number(s) or give detail |
| --- | --- | --- |
| **Word count** | **The paper is 7000 words or fewer** | ✓ |
| **Abbreviations** | **My paper does not contain abbreviations.**  Avoid using abbreviations, acronyms or “initialisms” anywhere in the paper other than SI units, common statistical terms. Abbreviations may be used in tables where they should be defined in a footnote. | ✓ |
| **Reporting guideline** | **The paper has been prepared using a recognized reporting guideline appropriate to the method / type of paper. Please identify the reporting guideline used in the box to the right.**  Please consult <https://www.equator-network.org/> to help select an appropriate guide [research and reviews only]. A checklist linked to the reporting guideline should be completed and uploaded as part of your submission. *If there is no applicable guideline, upload a blank file with the words ‘not applicable’ when requested at submission.* | ✓ |
|  | **My paper does not refer to *reporting* guidelines as a source for *methods.***  Statements such as “This trial was conducted according to the CONSORT guideline” **must not** be included | ✓ |
| **Title** | **The title is in the format ‘Topic / question: design/type of paper’** [not applicable to letters / editorials] | ✓ |
| **Abstract** | **The paper includes a structured abstract of no more than 400 words**  Use headings appropriate to the design of the study. [not applicable to letters / editorials] | ✓ |
|  | **No references are cited in the abstract.** | ✓ |
| **Study registration** | **The abstract includes detail of the study registration including number, the public registry**  **(e.g. ISRCTN), the registration date and date the first participant was recruited.**  If not registered, state ‘not registered’*[[1]](#footnote-2) [not applicable to narrative reviews and discussion papers, editorials, letters]. | ✓ |
|  | **For clinical trials (as defined by the ICMJE), registration occurred before the first participant was recruited.**  We are unable to consider clinical trials that were registered retrospectively – if uncertain check with editor before submission. | N/A |
| **Key words** | **I have included 4-10 keywords**  Use the Medical Subject Headings or Cumulative Index to Nursing and Allied Healthterms (see <http://www.nlm.nih.gov/mesh/meshhome.html>). | ✓ |
| **Contribution of the Paper statements** | **I have included up to 5 contribution statements.**  Under the headings “What is already known” and “What this paper adds” give 2 or 3 (maximum) short, single sentence bullet points (each) summarising key contributions. No references are to be cited. [not applicable to letters / editorials] | ✓ |
| **Multiple publications** | **I have explicitly mentioned other publications from the same study in my paper**  Other published and in press accounts of the study from which data in this paper originate are referred to in the paper (author details can be redacted for review if desired) and the relationship between this and other publications from the same study is made clear in the paper. see below] | ✓ |
|  | **I have provided references to any publications from the same study at the end of this checklist.** | ✓ |
| **Ethical approval and informed consent** | **I have given details of the ethical approval, including the body that granted it and reference number are iat the end of my methods section.**  This should include confirmation of informed consent by participants and / or elaboration of the basis for any exception. [**r**esearch papers only NA for reviews and discussion papers]. | ✓ |
| **Statistical reporting** | **I have followed journal guidance on reporting statistics, confidence intervals and statistical significance (summarizsd below)** | ✓ |
|  | - Exact p-values are stated to an appropriate degree of precision (typically no more than 3 decimal points). | ✓ |
|  | - The corresponding measure of effect or association and confidence interval are reported with all significance tests (including in the abstract). Confidence intervals can be used as the basis for inference without reference to statistical significance & ‘p-values’. | ✓ |
|  | - The term ‘statistically significant’ (not just ‘significant’) is used to refer to the result of tests. | ✓ |
|  | - p-values>0.05 are not to be used to conclude that there is no effect/association. | ✓ |
|  | Please consult Griffiths, P., Needleman, J., 2019. Statistical significance testing and p-values: Defending the indefensible? A discussion paper and position statement. International Journal of Nursing Studies 99, 103384. <https://doi.org/10.1016/j.ijnurstu.2019.07.001> for our policies on reporting point estimates, confidence intervals and p-values / significance tests & Lang, T.A., Altman, D.G., 2015. Basic statistical reporting for articles published in Biomedical Journals: The “Statistical Analyses and Methods in the Published Literature” or the SAMPL Guidelines. International Journal of Nursing Studies 52 (1), 5-9. <https://doi.org/10.1016/j.ijnurstu.2014.09.006> for more general guidance on statistical reporting |  |
| **Qualitative findings** | **Key quotations to support inferences are given with give meaningful (anonymous) individual subject identifiers for each quotation used.** | N/A |
| **Conflict of interests** | **I have stated any actual or potential conflicts of interest in a section at the end of the paper.**  If there are none, include a statement “Conflicts of interest: none”. The substance of this declaration should match details provided in file(s) uploaded at submission. | ✓ |
| **Please provide below references for any other publications based on data from the same study, and describe the relationship to the current study (see ‘multiple publications’, above).**  e.g. “A previous publication from this study showed that carrots were effective in preventing blindness (reference below). This study reports on a range of other health benefits hat were not included in that other study.”  *To assist editors, upload copies of papers where the abstract / full text is not readily available (including those under review elsewhere, which will be treated in strict confidence).*  *Where your paper is based on analysis of a publically available data set or is part of a series of publications from a large cohort study (or similar) you can be selective in the references you provide and give a more general account of how this paper relates to others but it is essential that editors are able to verify the unique contribution of the paper you are submitting.*  *If unsure about declarations we encourage you to err on the side of openness and suggest you consult Norman, I., Griffiths, P., 2008. Duplicate publication and 'salami slicing': Ethical issues and practical solutions. International Journal of Nursing Studies 45 (9), 1257-1260.*  **Do NOT use this space to copy your paper’s reference list.** | | |
| The questionnaire data from the present study was collected as part of a project evaluating the effectiveness of the Norwegian Labour Inspection Authority’s regulatory tools. The protocol paper for the project is referenced in the methods section.  Indregard et al. (2019). Effectiveness of the Labour Inspection Authority’s regulatory tools for work environment and employee health: study protocol for a cluster-randomised controlled trial among Norwegian home-care workers. *BMJ Open*, 9.  A previous publication concluded that the regulatory tools had no effect on psychosocial and mechanical work factors.  Garshol et al. (2022). Effects of the Labour Inspectorate Authority’s regulatory tools on psychosocial and biomechanical work factors in Norwegian home care services: a cluster randomised controlled trial. *Occup. Environ Med*. 0, 1-9.  A previous publication concluded that labor inspections and guidance-through-workshops had no statistically significant effect on self-reported health and physician-certified sick leave.  Garshol et al. (2023). Effects of the Labour Inspection Autority’s regulatory tools on physician-certified sick leave and employee health in Norwegian home-care services – a cluster randomized controlled trial. *Scand J Work Environ Health,* 50(1), 28-38.  A previous publication concluded that psychosocial work factors increased the risk of sick leave due to common mental disorders.  Knutsen et al. (2024). Impact of psychosocial work factors on risk of medically certified sick leave due to common mental disorders: a nationwide prospective cohort study of Norwegian home care workers. *BMC Public Health,* 24.  A previous publication concluded that leadership behaviors buffered the association between emotional dissonance and mental distress.  Johannessen et al. (2024). Emotional dissonance and mental health among home-care workers: A nationwide prospective study of the moderating role of leadership behaviors. *Scand J Work Environ Health,* online first. | | |

*© Peter Griffiths, Ian Norman 2021-2024 V2024.1*

*This work is licensed under a*[Creative Commons Attribution 4.0 International License.](http://creativecommons.org/licenses/by/4.0/)

1. While the journal endeavours to maintain a double blind-review process as far as possible, we give priority to transparent reporting and prospective registration. As it is important that reviewers are able to verify that reporting is complete and consistent with protocols to avoid (for example) selective outcome reporting or undocumented protocol changes, authors are not permitted to redact registration numbers for review. [↑](#footnote-ref-2)
